# Chalkophomycin Biosynthesis Revealing Unique Enzyme Architecture for a Hybrid Nonribosomal Peptide Synthetase and Polyketide Synthase

**DOI:** 10.3390/molecules29091982

**Published:** 2024-04-25

**Authors:** Long Yang, Liwei Yi, Bang Gong, Lili Chen, Miao Li, Xiangcheng Zhu, Yanwen Duan, Yong Huang

**Affiliations:** 1 Department of Immunology, School of Basic Medical Sciences, Anhui Medical University, Hefei 230032, China; yl2420841262@163.com; 2 Hefei Comprehensive National Science Center, Institute of Health and Medicine, Hefei 230093, China; lily@ihm.ac.cn; 3 Xiangya International Academy of Translational Medicine, Central South University, Changsha 410013, China; yiliwei318@usc.edu.cn (L.Y.); gongbang025@163.com (B.G.); 17388946043@163.com (M.L.); seanzhu@aliyun.com (X.Z.); ywduan66@sina.com (Y.D.); 4 Department of Pharmacy, The Affiliated Nanhua Hospital, Hengyang Medical School, University of South China, Hengyang 421001, China; 5 College of Pharmacy, Hunan Vocational College of Science and Technology, Changsha 410004, China; 6 Hunan Engineering Research Center of Combinatorial Biosynthesis and Natural Product Drug Discovery, Changsha 410011, China; 7 National Engineering Research Center of Combinatorial Biosynthesis for Drug Discovery, Changsha 410011, China

**Keywords:** chalkophore, copper, peptide, hybrid NRPS/PKS, chain release mechanism, reductase domain (R^0^)

## Abstract

Chalkophomycin is a novel chalkophore with antibiotic activities isolated from *Streptomyces* sp. CB00271, while its potential in studying cellular copper homeostasis makes it an important probe and drug lead. The constellation of *N*-hydroxylpyrrole, 2*H*-oxazoline, diazeniumdiolate, and methoxypyrrolinone functional groups into one compact molecular architecture capable of coordinating cupric ions draws interest to unprecedented enzymology responsible for chalkophomycin biosynthesis. To elucidate the biosynthetic machinery for chalkophomycin production, the *chm* biosynthetic gene cluster from *S*. sp. CB00271 was identified, and its involvement in chalkophomycin biosynthesis was confirmed by gene replacement. The *chm* cluster was localized to a ~31 kb DNA region, consisting of 19 open reading frames that encode five nonribosomal peptide synthetases (ChmHIJLO), one modular polyketide synthase (ChmP), six tailoring enzymes (ChmFGMNQR), two regulatory proteins (ChmAB), and four resistance proteins (ChmA′CDE). A model for chalkophomycin biosynthesis is proposed based on functional assignments from sequence analysis and structure modelling, and is further supported by analogy to over 100 *chm*-type gene clusters in public databases. Our studies thus set the stage to fully investigate chalkophomycin biosynthesis and to engineer chalkophomycin analogues through a synthetic biology approach.

## 1. Introduction

**Figure 1:**
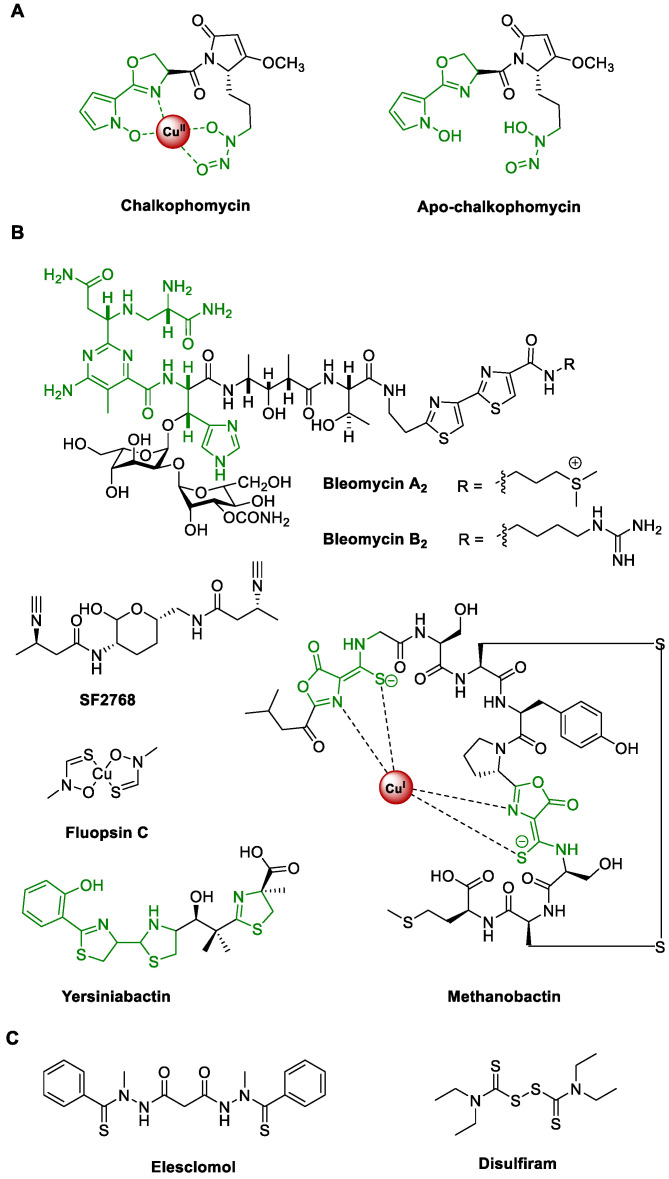
Representative natural and synthetic chalkophores. (**A**) the structures of chalkophomycin and apo-chalkophomycin; (**B**) the structures of selected natural chalkophores; (**C**) synthetic chalkophores with interesting biological activities.

Chalkophomycin, originally isolated from *Streptomyces* sp. CB00271 in 2021, is an unprecedented copper(II)-binding metallophore (Figure 1A) [1]. The gross structure of chalkophomycin was deduced by single-crystal X-ray analysis, and its X-ray photoelectron spectroscopy analysis revealed that Cu(II) is the majority of copper species. The structure of the copper-less apo-chalkophomycin was established by spectroscopic analysis, and its absolute stereochemistry was based on the similar circular dichroism spectra with chalkophomycin. These analyses revealed that Cu(II) in chalkophomycin is coordinated to *N*-hydroxylpyrrole, 2*H*-oxazoline, and diazeniumdiolate from a methoxypyrrolinone ring. These respective functional groups in chalkophomycin have been found in dozens of natural products, e.g., glycerinopyrin, hormaomycins, and surugapyrroles (for *N*-hydroxylpyrrole) [2,3,4], coelibactin, mycobactin, and aerucyclamides (for 2*H*-oxazoline) [5,6], alanosine, fragin, and gramibactin (for diazeniumdiolate) [7,8,9,10,11,12], and althiomycin, dolastatin 15, and malyngamide A (for methoxypyrrolinone) [13,14,15,16]. However, compact integration of these distinct elements into one molecular architecture capable of coordinating cupric ions highlights nature’s unique design strategy for chalkophores, an emerging family of natural products responsible for microbial copper homeostasis [17].

In an analogy to siderophores for iron homeostasis, there is growing interest to study the biological function and biosynthesis of chalkophores [18,19,20,21,22,23,24,25,26]. Chief among them are members of the methanobactin family, which were first identified from methane-oxidizing bacteria. These methanotrophic bacteria produce an abundant amount of copper enzyme named particulate methane monooxygenase, which catalyzes the aerobic oxidation of methane and plays an indispensable role in the global carbon cycle. Recent genome mining efforts have revealed that some other bacteria may also produce methanobactins for copper acquisition [19]. A wider range of bacteria can produce chalkophores, including bleomycin, yersiniabactin, SF2768, and xanthocillins (Figure 1B). Interestingly, yersiniabactin was initially discovered as a siderophore, but its noncanonical role for copper and other non-iron metal ion uptake was recently discovered in pathogenic *Enterobacteriaceae*, supporting intricate interactions between host and pathogens, mediated by natural products and transition metal ions [27,28]. Copper can not only serve as an active site cofactor for certain proteins, e.g., “blue” copper proteins, and the aforementioned particulate methane monooxygenase, but also regulate protein function allosterically in signaling pathways in cancer, fatty liver disease, neurodegeneration, and obesity [29]. Therefore, methanobactins have been used in the treatment of acute Wilson’s disease in a WD rat model to alleviate copper overload, since excess copper causes hepatocyte death [30]. In addition, cuproptosis, a new form of cell death targeting lipoylated TCA cycle proteins, was recently discovered using elesclomol, a synthesized chalkophore (Figure 1C) [31]. Combination treatment with copper and disulfiram, an old drug against alcohol abuse, also showed promise to induce cancer cell cuproptosis [32]. Taken together, these copper-binding molecules represent interesting drug leads and powerful small-molecule probes to elucidate the roles of copper-signaling pathways.

The purpose of our study was to discover and characterize the chalkophomycin biosynthetic gene cluster (*chm*). The long-term goal of our study focused on chalkophomycin is to develop novel probes for cuproptosis and cuproplasia, and potential drug leads for the treatment of cancer and Wilson’s disease. We report here on (i) the discovery and genetic characterization of the *chm* gene cluster in *S*. sp. CB00271; (ii) bioinformatics analysis of the *chm* cluster and a biosynthetic proposal for chalkophomycin biosynthesis involving a hybrid nonribosomal peptide synthetase and polyketide synthase (NRPS/PKS); and (iii) genome mining of the *chm* pathway revealing its global distribution in a wide range of actinomycetes. Our study now enables rapid access to chalkophomycin gene clusters, as well as genome mining of individual biosynthetic enzymes for the formation of *N*-hydroxylpyrrole, 2*H*-oxazoline, diazeniumdiolate, and methoxypyrrolinone. The stage is now set for a synthetic biology approach to engineer chalkophomycin analogues as small-molecule probes, drug leads, and potential chiral transition metal catalysts.

## 2. Results and Discussion

### 2.1. Discovery and Genetic Characterization of the chm Gene Cluster in S. sp. CB00271

**Figure 2:**
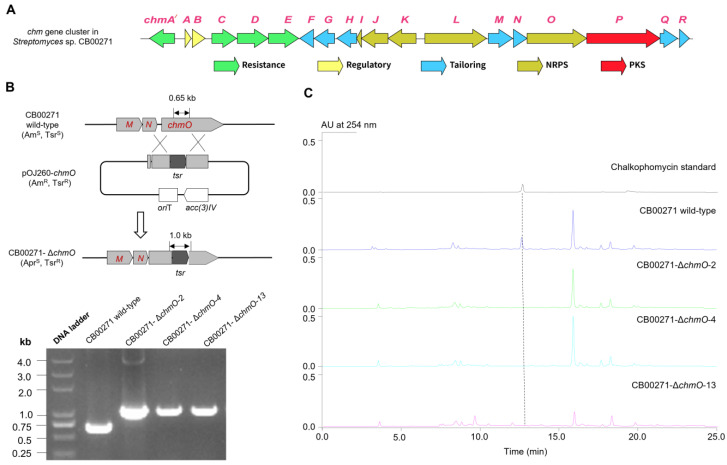
Identification and confirmation of the *chm* gene cluster. (**A**) The *chm* gene cluster contains a total of 19 ORFs from *chmA′* to *chmR*; (**B**) Gene replacement of *chmO* NRPS gene by a thiostrepton-resistant gene; (**C**) HPLC analysis revealed that the three *S*. sp. CB00271::Δ*chmO* mutants all abolished the production of chalkophomycin, in comparison to the wild-type strain.

The *chm* gene cluster was discovered by genome mining of putative biosynthetic genes in *S*. sp. CB00271 responsible for diazeniumdiolate and methoxypyrrolinone formation (Figure 2). There have been over 300 nitrogen–nitrogen-bond-containing natural products discovered, which bear a variety of important functional groups, e.g., diazo, hydrozones, pyrazole, and diazeniumdiolate [33]. Pioneering studies of diazeniumdiolate biosynthesis in streptozotocin, l-alanosine, and fragin/valdiazen revealed multiple unique enzymes *en route* for *N*–*N* construction and further morphing from amino acid precursors, including SnzE/SznF, AlnDEFGLMN, and HamACED/HamACEDG [3,7,8,9,34,35]. In particular, Hertweck and co-workers recently discovered that GrbED and their homologs are responsible for the biosynthesis of l-graminine, an unnatural amino acid found in a handful of cyclic or linear peptides, such as gramibactin, gladiobactin, JBIR-141/JBIR-142, megapolibactins, plantaribactin, and trinickiabactins [10,11]. These l-graminine-containing peptides are ubiquitously assembled by NRPSs, during which, l-graminine is activated by specific adenylation domains and loaded onto the thiolation domain in the NRPS assembly line. We therefore hypothesized that the diazeniumdiolate biosynthesis and loading in chalkophomycin is likely to follow the similar logic for the biosynthesis of gramibactin and the like, in which a similar analogous enzyme pair of GrbED should be present in the *chm* gene cluster. Therefore, using GrbD and GrbE as query sequences, we identified three sets of GrbD and GrbE homologous genes from the genome of *S*. sp. CB00271 (Figure S1). Sequence alignment revealed that they share 33–35% sequence identity with GrbE, and 38% sequence identity with GrbD. AntiSMASH-based analysis predicted that they are located in the flanking regions of several NRPSs in the genome of *S*. sp. CB00271, similar to the gramibactin gene cluster in *Paraburkholderia graminis* [10,36].

In addition, the methoxypyrrolinone moiety in chalkophomycin is also found in several other natural products, including althiomycin, dolastatin 15, sintokamide A, malyngamide A, and mirabimide E [13,14,37,38]. A malonyl-specific PKS module and a standalone *O*-methyltransferase were proposed for methoxypyrrolinone biosynthesis in althiomycin (Figure 2B) [13,14]. Therefore, close examination of the flanking regions of GrbE and GrbE homologs revealed that one cluster has a predicted PKS (WP_073800092.1) and an *O*-methyltransferase (WP_073800093.1). This PKS shows 42% sequence identity with module six of AlmB for althiomycin, while the *O*-methyltransferase shows 42% sequence identity with PokM3, responsible for *O*-methylation in pikromycin biosynthesis (Figure S2) [39]. Therefore, this gene cluster was named the chalkophomycin biosynthetic gene cluster (*chm*). In contrast, there are no such PKS and *O*-methyltransferase genes in the other two gene clusters.

The overall GC content of the *chm* gene cluster is 72%, similar to other *Streptomyces* DNA. Bioinformatics analysis of the *chm* cluster revealed 19 open reading frames (ORFs) (Figure 2B). Comparison of the deduced gene products from the *chm* gene cluster with proteins of known functions in the database facilitated the functional assignment of individual ORFs (Table 1). While this manuscript was in preparation, the same *chm* cluster from *Streptomyces* sp. CB00271 was reported to be responsible for chalkophomycin biosynthesis based on genome mining of SznF for streptozotocin biosynthesis, albeit without *chmA′* and *chmR*; however, gene replacement of this cluster was not performed [40].

In order to study whether *chmO* encodes an NRPS from the *chm* gene cluster that is involved in the biosynthesis of chalkophomycin in *S*. sp. CB00271, a 546 bp DNA fragment inside of *chmO* was replaced by a mutant copy in which *chmO* was disrupted by the thiostrepton-resistant gene with a *kas*Op* promoter (Figure 2B) [41]. The gene replacement of *chmO* completely abolished the production of chalkophomycin in *S*. sp. CB00271 (Figure 2C). This result suggests that the identified *chm* gene cluster in *S*. sp. CB00271 is responsible for chalkophomycin biosynthesis.

### 2.2. Bioinformatics Analysis of the chm Cluster in S. sp. CB00271 Revealed a Hybrid NRPS/PKS for Chalkophomycin Biosynthesis

#### 2.2.1. Overview of the *chm* Gene Cluster

The *chm* gene cluster encompasses 19 ORFs designated *chmA′* to *chmR* (Figure 2A and Table 1). These biosynthetic genes encode NRPSs (ChmI, ChmJ, ChmK, ChmL, ChmO), PKS (ChmP), and other tailoring enzymes (ChmF, ChmG, ChmH, ChmM, ChmN, ChmQ, ChmR); among them, two are regulatory genes (ChmA and ChmB), and four are resistance genes (ChmA′, ChmC, ChmD, and ChmE).

#### 2.2.2. Biosynthesis of NRPS Precursors *N*-Hydroxylpyrrole and l-Graminine

**Figure 3:**
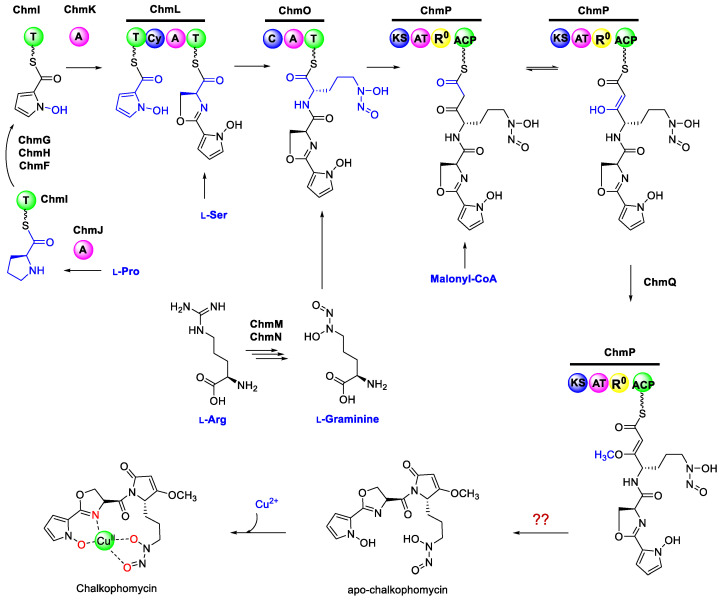
Chalkophomycin is proposed to be biosynthesized by a hybrid NRPS/PKS. The biosynthesis of chalkophomycin may start from the formation of ChmI-*N*-hydroxypyrrole through the enzymatic actions of ChmFGHJ. The standalone ChmK adenylation enzyme and ChmL NRPS may mediate the transfer of the *N*-hydroxypyrrole moiety, followed by the generation of 2*H*-oxazoline. ChmMN may be responsible for the biosynthesis of l-graminine from l-Arg, which would be adenylated and loaded to ChmO NRPS. ChmP PKS may catalyze a two-carbon elongation to construct the full-length peptidyl–polyketide chain. Subsequent tailoring steps involving ChmQ methyltransferase and the ChmP_R^0^ domain may furnish the five-membered methoxypyrrolinone for the release of apo-chalkophomycin, followed by the chelation of a cupric ion to yield chalkophomycin.

Although pyrrole is found in a number of natural products biosynthesized by NRPSs, including clorobiocin, cloumermycin A1, plyoluteorin, prodigiosin, and undecylprodigi-osin, the *N*-hydroxylpyrrole building block is rare in nature. Considering that the formation of some pyrroles is catalyzed by a four-electron, two-step process from proteinogenic amino acid l-proline mediated by FAD-dependent reductases [42,43,44], biosynthesis of *N*-hydroxylpyrrole may adapt a similar route to generate a *N*-pyrrolyl-2-thioester-peptidyl carrier protein (PCP), followed by *N*-oxidation *en route* to *N*-hydroxylpyrrolyl-2-carboxyl-S-PCP. Careful examination of the *chm* gene cluster revealed a small “*N*-hydroxylpyrrole” gene cassette containing *chmGHIJK*, which is putatively responsible for *N*-hydroxylpyrrole biosynthesis in chalkophomycin (Figure 3). The identified genes were as follows: (1) a free-standing 50 kDa l-proline-specific adenylation (A) domain ChmJ responsible for l-proline activation to form l-prolyl-AMP; (2) the free-standing PCP ChmI would be loaded with l-prolyl-AMP to form l-pyrolyl-*S*-ChmI; (3) a predicated flavoprotein ChmH is presumably responsible for oxidizing pyrolyl-*S*-ChmI to pyrrolyl-*S*-ChmI, since it resembles CloN3, an l-prolyl-PCP dehydrogenase for pyrrole biosynthesis in antibiotic clorobiocin [43]; and (4) another flavoprotein, ChmG, may catalyze pyrrolyl-*S*-ChmI oxidation to form *N*-pyrrolyl-*S*-ChmI. ChmF is predicated to be a proline iminopeptidase, sharing 24% and 37% sequence identity and similarity to AlmF in althiomycin biosynthesis in *M. xanthus* DK897, respectively, which is proposed for its methoxylpyrrolinone formation. However, the role of ChmF in *N*-hydroxylpyrrole formation or methoxylpyrrolinone biosynthesis in chalkophomycin remains to be determined.

Biosynthesis of an l-graminine monomer in chalkophomycin may involve the enzymatic action of ChmM and ChmN, since they shared 35% and 37% sequence identity with GrbE and GrbD, respectively. Synthetic l-graminine could restore the production of gramibactin in individual ΔGrbE and ΔGrbD mutants in *P. graminis* [11]. Therefore, l-graminine may be biosynthesized from l-Arg by ChmM and ChmN, followed by incorporation into the chalkophomycin assembly line in *S*. sp. CB00271. This is also consistent with the recent observation by Bulter and co-workers that the unnatural amino acid l-graminine is derived from l-Arg in gramibactin biosynthesis, by isotopic labeling studies [12].

#### 2.2.3. Chalkophomycin Biosynthesis by a Hybrid NRPS/PKS

After the conversion of l-Pro to pyrrolyl-*S*-ChmI catalyzed by ChmFGHIJ, a free-standing adenylation enzyme, ChmK, may mediate the transfer of the *N*-pyrrolyl intermediate to the first PCP domain on ChmL NRPS. ChmL is an NRPS with the characteristic PCP-Cy-A-PCP domain organization, in which the Cy (cyclization) domain is responsible for heterocycle formation in NRPS assembly lines, while the A domain in ChmL is predicted to activate l-cysteine. We proposed that this A domain is responsible for biosynthesis of the oxazoline moiety of chalkophomycin by loading l-serine to its cognate PCP; albeit, further biochemical confirmation is needed. In the PCP domain of ChmO NRPSs and the discrete ChmI PCP with the signature motif of Gx(H/D)S, the Ser needs be modified through the covalent attachment of the 4-phosphopantetheine group.

Following the ChmL NRPS is the ChmO protein with the characteristic NRPS condensation C-A-PCP domain organization, which may be responsible for activating and loading l-graminine to its cognate PCP domain, and the C domain may catalyze its condensation with the upstream dipeptidyl intermediate. Although the A domain in ChmO is predicted to activate l-*N*-hydroxyformyl ornithine, it is likely responsible for loading the unnatural l-graminine to chalkophomycin assembly line, since it also resembles the A domains for l-graminine activation in several NRPSs for the biosynthesis of megapolibactins and gladiobactin/plantaribactin with 30~34% sequence identities (Figure S3) [11].

**Figure 4:**
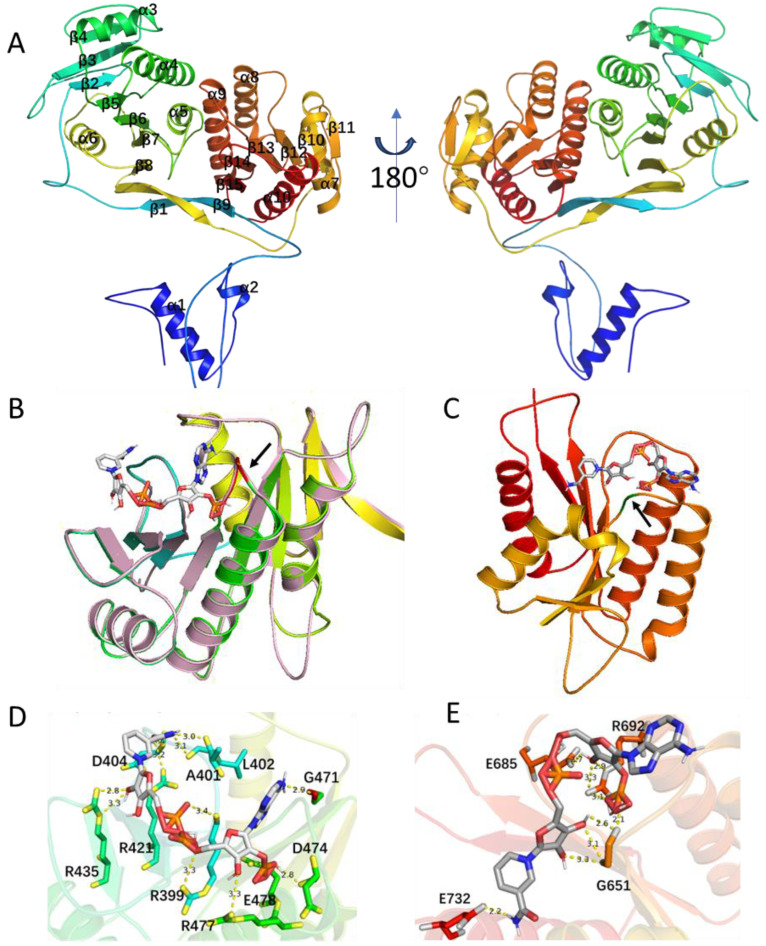
Structural analysis of ChmP_R^0^ domain with two putative Rossmann-like motifs (RLM). (**A**) AlphaFold2-generated model of the ChmP_R^0^ domain from *S*. sp. CB00271. (**B**,**C**) Docking analyses of the binding modes of NADP(H) to the ChmP_R^0^ domain from *S*. sp. CB00271 using AutoDock, respectively. The loop sequence marked in red is GSAAGPA in (**B**), while the loop sequence marked in green is GGG in (**C**). The RLM in MUN77 is colored pink in (**B**). (**D**,**E**) The potential interaction of NADPH with R^0^ domain RLMs. Amino acid residues of RLMs involved in the NADP(H) interaction are shown as sticks. Distance between NADP(H) and potential binding amino acids are indicated as yellow dashed lines.

The *chmP* gene encodes a protein of 1414 amino acid residues containing one ketosynthase (KS), one malonyl-specific acyltransferase (AT), one domain with potential reduction function (named as R^0^) (amino acid residues 823–1299), and one acyl carrier protein (ACP). The KS is highly homologous to typical KSs from hybrid PKS/NRPS (Figure S3) [45,46,47,48], including EpoC for epothilone biosynthesis (Figure S4) [49]. The KSs in the hybrid NRPS/PKS would be responsible for transferring the dipeptidyl intermediate from the ChmO PCP domain, and they catalyze the condensation with the incoming malonyl-ACP, mediated by its only AT domain. The AT-R^0^ didomain in ChmP shares 27% sequence identity with LnmG, a well-characterized, free-standing AT-oxidoreductase didomain protein for leinamycin biosynthesis [46]. AlphaFold2 [50] prediction further reveals that the ChmP_R^0^ domain might contain two Rossmann-like motifs (RLM) (Figure 4).

We first employed Alphafold2 to predict the structure of the ChmP_R^0^ domain, which displays an alternating pattern of β-sheet–α-helix–β-sheet (Figure 4A). At the N- and C-terminus of ChmP_R^0^, there exists a double-wound three-layer α/β/α sandwich topology (Figure 4A). The N-terminal region features a typical Rossmann fold with a central β-sheet (strands β2–β7) arranged in the order 321456 (Figure 4A). Furthermore, this segment bears high similarity to the NADB region of the ChmP homologous protein from *Steptomyces* sp. MUN77 in both sequence and structure (Figure 4B and Figure S5). The C-terminal includes a minimal RLM that crosses between the second and third strands in the order of 213456, and it is sandwiched between a layer of α-helices (Figure 4A) [51]. These two RLMs are connected by a pair of antiparallel β-sheets.

The RLM usually possesses binding capability for diphosphate-containing cofactors such as NADP(H). The N-terminal turn of the first α-helix in RLM often binds to phosphate, and the gap between the first and third β-strands, β1 and β3, formed by the cross, could accommodate larger substrates or cofactors [52]. Accordingly, NADP was docked into both conservative pockets of RLM in the ChmP_R^0^ domain using an AF2 prediction structure through AutoDock Vina [53]. The docking of NADP into the binding pocket of RML at the N-terminus of ChmP_R^0^ resulted in nine conformers with affinities ranging from −7.7 to −8.1 kcal/mol, while RML at the C-terminus of ChmP_R^0^ had affinities ranging from −6.8 to −7.1 kcal/mol. These docking analyses revealed that NADP can potentially bind to the cofactor binding pocket of both RLMs in the ChmP_R^0^ domain. As is shown in Figure 4B,C, the structure with the highest binding free energy score was chosen for visualization. Additionally, each RLM in the ChmP_R^0^ domain contains a Gly-rich loop located in the C-terminal end of the β-sheet at termination of the crossover (indicated by the black arrow), which may become a part of RLM active site. The analysis of the putative NADPH binding sites revealed that NADPH can not only establish extensive polar interactions with both acidic and basic amino acids in the R^0^ domain pocket, but also potentially engage in interactions with glycine within the Gly-rich loop (Figure 4D,E).

In the biosynthesis of cyclopiazonic acid in *Aspergillus* sp., a reductase-like R* domain in the C-terminal of CpaS can carry out Dieckmann cyclization in a non-redox fashion [54]. Therefore, the R^0^ domain in ChmP may subtract one hydrogen from the ACP-tethered ketoamide, which leads to the formation of a resonance-stabilized carbanion that undergoes *O*-methylation through ChmQ. Alternatively, the tethered ketoamide tautomerizes and the resulting enol form could be methylated by ChmQ. Intriguingly, ChmP PKS lacks a type I thioesterase domain for acyl-ACP intermediate release upon completion of chain elongation in modular PKSs [55,56]. The release of the peptidyl-acyl chain and the formation of an amide in the methoxypyrrolinone moiety may be executed non-enzymatically. During althiomycin biosynthesis in *M. xanthus* DK897 and an insect pathogen *Serratia marcescens* Db10, an iminopeptidase AlmF or a type II thioesterase Abl6 is proposed to play certain roles for chain release, respectively [13,14]. Therefore, it remained to be determined whether the putative proline iminopeptidase ChmF may also facilitate chalkophomycin release.

Chalkophores are relatively rare in nature compared to siderophores, and the prototypical chalkophore methanobactins are biosynthesized ribosomally and then undergo extensively morphing [17,18,19]. In contrast, chalkophomycin biosynthesis follows the assembly model of hybrid NRPS/PKS. In most characterized NRPS/PKSs, these mega-synthetases/synthases often encompass multiple modules, with typical domain organization of C–A–PCP in a NRPS module and KS–AT–(KR–DH)–ACP in a PKS module. There are extensive intra- or intermodular communications mediated by conserved linkers responsible for the efficient transfer of peptidyl- or acyl-intermediates. The assembly line models were studied using several model systems, e.g., DEBS for PKSs, and surfactin and tyrocidine for NRPSs [57,58]. However, the complex module organization of these huge mega-synthases poses formidable challenges to isolate, purify, and characterize them biochemically and structurally [59]. ChmL/ChmO/ChmP are all single-module proteins, and thus provide a rare opportunity to study their inter-modular communications, in particular, how the peptidyl-*S*-PCP from ChmL was transferred to ChmP KS. 

#### 2.2.4. Regulatory and Resistance Genes for Chalkophomycin Biosynthesis

There are two putative regulatory genes in the *chm* gene cluster, including *chmA* and *chmB*. ChmA gene encodes a regulatory LuxR family protein, which shows 35% sequence identity with RimR2, a recently identified positive pathway-situated regulator from *Streptomyces rimosus* M527 for rimocidin biosynthesis [60]. The ChmB gene encodes a TetR/AcrR-like transcription regulator, which shows 32% sequence identity with SCO1718 from *Streptomyces coelicolor*. Four genes, *chmA′*, *chmC*, *chmD*, and *chmE*, could be identified within the *chm* gene cluster, which encode gene products to confer putative resistance to chalkophomycin. Both ChmA′ and ChmC show 34% or 32% sequence identity with EfpA, a well-characterized multi-drug efflux pump from *Mycobacterium tuberculosis* [61]. ChmD and ChmE encode a pair of ABC transporters, presumably to form an ATP binding cassette transporter complex responsible for chalkophomycin efflux.

### 2.3. Chalkophomycin-Type Gene Clusters Are Wide-Spread among Diverse Bacterial Strains

**Figure 5:**
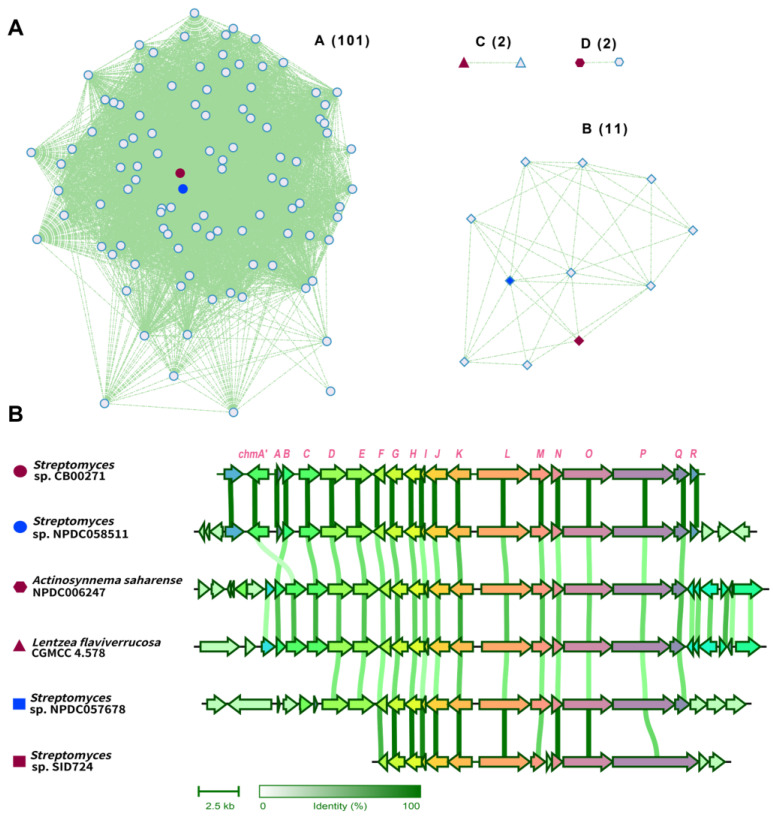
The global distribution of *chm* gene cluster in a wide range of actinomycetes. (**A**) Analysis of 116 *chm*-type gene clusters. (**B**) Representative *chm*-type gene clusters.

Homologous *chm* gene clusters were first identified from GenBank, based on a cblaster [62] search using genes including *chmA′* to *chmR*, and Blastn [63] search using *chmP* as a query sequence; after manual removal of identical gene clusters, this search resulted in 39 homologous *chm* gene clusters (Figure 5 and Figure S6). With the availability of 11,357 assembled genomes in the Natural Products Discovery Center at UF Scripps Research [64], a ChmP-based BlastP search further resulted in 209 candidate gene clusters from 196 bacterial genomes. Close examination of the ChmP homologs in these candidate clusters suggested that the gene clusters with an identity cutoff of 50% for its ChmP homolog would result in 77 *chm*-type gene clusters. Next, *chm* and a total of 116 of these *chm*-type gene clusters were analyzed by BIG-SCAPE [65], and their gene cluster similarity network was constructed and visualized using Cytoscape [66] (Figure 5A). The majority of these gene clusters were closely clustered to *chm* gene clusters with 101 members. The other three gene cluster families contain 11, 2, and 2 members.

Representative gene clusters from each cluster family were selected and aligned (Figure 5B). The *chm*-type gene cluster from *Streptomyces* sp. NPDC057678 is highly homologous to the *chm* gene cluster from *S*. sp. CB00271, showing >95% sequence identity across *chmA′* to *chmR.* In both *chm*-type gene clusters in rare actinomycetes, e.g., *Actinosynnema saharense* NPDC006247 and *Lentzea flaviverrucosa* CGMCC4.578, the multi-drug transporter EmrB/QacA is instead positioned downstream of two genes encoding a TetR transcription regulator and a molybdenum cofactor sulfurase C-terminal domain protein, respectively. Furthermore, there are several notable differences in the *chm*-type gene cluster from *Streptomyces* sp. SID724, including (a) a significantly larger PKS (1914 a.a) with a KS–AT–dehydratase–reductase domain organization, (b) an additional small cupin-domain-containing protein, and (c) the lack of an *O*-methyltransferase. Therefore, a new chalkophore with distinct structure may be produced in this specific strain. Taken together, these analyses suggest that *chm*-type gene clusters are widely distributed in actinomycetes, especially in *Streptomyces*. It remained to be determined whether the presence of these gene clusters conveys a certain survival advantage for the host strain, since the essential cupric ions may be assimilated to the host through the produced chalkophores. Alternatively, chalkophomycin was also toxic to some tested bacteria, and the producing strain may also have an advantage over its competitor in the surrounding environment.

The availability of these *chm*-type gene clusters may provide additional possibilities to study chalkophomycin biosynthesis in an evolutionary context, as we have observed in the genome neighborhood analyses (Figure 5). These efforts may lead to the discovery of new enzymology for chalkophore biosynthesis and potential for a synthetic biology approach to discover and engineer chalkophores. Although copper homeostasis is essential for most life on earth, most actinomycetes are soil-dwelling bacteria. The identification of over 100 homologous *chm*-type gene clusters in not only *Streptomyces* species, but also in rare actinomycetes, i.e., *Actinosynnema saharense* and *Lentzea flaviverrucosa*, not only suggests the horizonal gene transfer of the gene clusters, but also implies the important role of chalkophomycin and the like for these microorganisms. Considering the discovery of several chalkophores from fungi and bacterial pathogens [17], understanding their physiological roles may be instrumental to study the role of copper ions in living organisms, including *Homo sapiens* [29].

## 3. Materials and Methods

### 3.1. General Experimental Procedures

All chemical and biological reagents were purchased from commercial sources unless otherwise specified. Chalkophomycin and crude extracts were analyzed by a Waters E2695 HPLC system equipped with a Welch AQ-C18 column (5 μm, 250 × 4.6 mm, Welch Materials Inc., West Haven, CT, USA) and detected with a photodiode array detector. Genomic DNA was isolated following standard protocols [67]. Plasmid DNA was extracted and purified using a PM0201 kit (Tsingke Biotech. Co., Beijing, China). The restriction endonucleases were purchased from New England Biolabs. DNA manipulation was based on standard procedures, including restriction endonuclease digestion and transformation.

### 3.2. Strains, Plasmids, and Culture Conditions

*Streptomyces* sp. CB00271 was preserved in our lab. For sporulation, all strains were grown at 30 °C on an R2A solid medium. *Escherichia coli* DH5α and S17-1 were used for cloning and intergeneric conjugation, respectively, and all were cultured with Luria–Bertani medium. All conjugants were grown on mannitol soya flour solid medium containing 10 mM MgCl_2_. For the cultivation of corresponding mutants, antibiotics including 50 mg/L apramycin, 25 mg/L thiostrepton, and 40 mg/L nalidixic acid were supplemented accordingly. All applied media are described in the Supplementary Materials. All strains and plasmids are listed in Table S1.

### 3.3. Fermentation Production and HPLC Analysis of Chalkophomycin

The spores of *Streptomyces* sp. CB00271 and its mutant strains were inoculated into Erlenmeyer flasks (250 mL) containing 50 mL of tryptic soy broth medium (1.7% tryptone, 0.3% soya peptone, 0.25% dextrose, 0.5% NaCl, 0.25% K_2_HPO4, pH 7.3) at 28 °C on a shaker at 230 rpm for 24~48 h, with or without the addition of antibiotics. Then, ~10% (*v*/*v*) seed cultures were transferred into 50 mL production medium (2% soluble starch, 2% soy bean flour, 0.05% KH_2_PO_4_, 0.025% MgSO_4_) in 250 mL Erlenmeyer flasks. The pH of the production medium was adjusted to 7.0, followed by the addition of 0.5% (*w*/*v*) CaCO_3_ and 8.0% (*v*/*v*) macroporous resins DA201-H (Jiangsu Su Qing Water Treatment Engineering Group Co., Ltd., Jiangyin, China). These *Streptomyces* strains were then cultured for 7 days on a shaker at 230 rpm/28 °C.

For HPLC analysis of chalkophomycin production, the mobile phase included buffer A (ultrapure H_2_O containing 0.1% HCO_2_H) and buffer B (chromatographic-grade CH_3_CN containing 0.1% HCO_2_H). A linear-gradient program (95% buffer A for 2 min; 95% buffer A to 5% buffer A for 20 min; 5% buffer A for 2 min; 5% buffer A to 95% buffer A for 1 min; followed by 95% buffer A for 2 min) was applied at a flow rate of 1 mL/min.

### 3.4. Gene Replacement of chmO in S. sp. CB00271

A pOJ260-based plasmid pXY5001 was constructed to generate the *ΔchmO* gene replacement mutant in *S*. sp. CB00271 via a double-crossover homologous recombination. To inactivate *chmO*, a 546 bp fragment of the *chmO* gene was replaced with the thiostrepton-resistance gene with a kasOp* promoter using the In-Fusion cloning kit (Tsingke, China), and the mutated *chmO* gene was cloned into pOJ260 between the *Hind*III and *Xba*I restriction sites. This plasmid was introduced into *Streptomyces* sp. CB00271 by conjugation and selected for thiostrepton resistance and the apramycin-sensitive phenotype to isolate the desired double-crossover mutant strains. The PCR primers are shown in Table S2.

### 3.5. Structural Analysis of the ChmP_R^0^ Domains in S. sp. CB00271

The ChmP_R^0^ domain in *S*. sp. CB00271 was predicted using AlphaFold2 [50]. Molecular docking was performed by AutoDock Vina, the predicted model of ChmP_R^0^ domains [53]. AutoDock Tools (The Scripps Research Institute, La Jolla, CA, USA) was used to prepare the ligands and receptor as pdbqt files after removing water, and adding polar hydrogen atoms and Gasteiger charges, respectively. The docking grid box size used was adjusted accordingly to encompass the NADP interaction site. Other default parameters were used. The best docking pose (most stable) was selected for binding mode comparison. The ligand–protein interaction structures were generated in PyMol (The PyMOL Molecular Graphics System, Version 3.0 Schrödinger, Inc., New York, NY, USA) [68].

### 3.6. Gene Cluster Similarity Network Analysis of chm Genes in Public Databases

In order to identify homologous *chm* gene clusters from GenBank, cblaster (version 1.3) was used to search for similar gene clusters from the nonredundant database in GenBank with a 50% sequence identity cutoff and the default parameters of 20,000 max intergenic gap [62]. These identified *chm*-like genes contain at least eight homologous genes from the identified *chm* gene cluster in *S*. sp. CB00271. In addition, a Blastn search with *chmP* as the probe (50% identity cutoff) was performed [63]. The identified gene clusters were manually checked to remove duplicated gene clusters. Similarly, similar *chm* gene clusters were also identified by BlastP search using ChmP from the Natural Product Discovery Center actinomycete genome database from the UF Scripps Research, using a 50% sequence identity cutoff [64]. BIG-SCAPE (version 1.1.5) was used to analyze these gene clusters with a default parameter cutoff of 0.3 [65]. The resulting data were visualized using the organic layout in Cytoscape (version 3.10) [66]. Clinker 0.0.28 was used to generate cluster comparisons when running in the Basic pipeline [69].

## 4. Conclusions

In this study, the biosynthetic gene cluster for chalkophomycin was identified from *S*. sp. CB00271, revealing an unusual hybrid NRPS/PKS with an atypical R^0^ domain in a PKS module, which might contain two RLMs for the binding of two NADP(H) cofactors. In addition, over 100 homologous *chm* gene clusters were discovered from public databases, suggesting the widespread nature of this gene cluster. Our study may help to unravel the evolutionary aspects of the chalkophomycin biosynthetic mechanism and potentially allow for the bioengineering of novel chalkophores that could serve as molecular probes and drug leads in the near future. The limitation of the current study is the lack of experimental validation of the functions of most assigned genes, while the chalkophomycin biosynthetic machinery provides a rare opportunity to study the interaction and evolution of NRPSs and PKSs through a multifaced approach using in vitro enzymatic assays, biophysical methods, and structural analyses.

## Figures and Tables

**Table 1:** Deduced Functions of Open Reading Frames in the Chalkophomycin Biosynthetic Gene Cluster.

Gene	Size (a.a.)	Putative Function	Protein Homologue	Identity%/Similarity%
*Orf(-2)*	293	Diacylglycerol kinase	WP_011029127.1	60/67
*Orf(-1)*	427	Adenylosuccinate synthase	4M0G_A	53/67
*ChmA′*	484	MFS transporter	EfpA (ALB20045)	34/53
*ChmA*	119	Regulatory LuxR family protein	RimR2 (QAS68949)	35/59
*ChmB*	248	TetR/AcrR-like transcription regulators	SCO1718 (CAB50933)	32/40
*ChmC*	478	MFS transporter	EfpA (ALB20045)	32/51
*ChmD*	595	ABC transporter	SCO7689 (CAC17506)	48/62
*ChmE*	582	ABC transporter	BDD77077	46/60
*ChmF*	264	Proline iminopeptidase	AlmF (CCA29204)	24/37
*ChmG*	395	Acyl-CoA/acyl-ACP dehydrogenase	TdaE (WP_014881725.1)	25/42
*ChmH*	383	l-prolyl-PCP dehydrogenase	CloN3 (AAN65232)	31/41
*ChmI*	92	Peptidyl carrier protein	CloN5 (AAN65234)	26/54
*ChmJ*	517	Adenylation protein	CloN4 (AAN65233)	31/48
*ChmK*	543	Adenylation protein	EntE (CAD6013920)	40/56
*ChmL*	1189	Non-ribosomal peptide synthetase	AlmA (CCA29202)	33/48
*ChmM*	469	l-graminine biosynthesis	GrbE (WP_006051176.1)	35/48
*ChmN*	264	l-graminine biosynthesis	GrbD (WP_006051175.1)	37/51
*ChmO*	1152	Non-ribosomal peptide synthetase	AlmA (CCA29202)	37/53
*ChmP*	1414	Type I polyketide synthase	AlmB (CCA29203)	42/54
*ChmQ*	339	O-methyltransferase	PokMT3 (ACN648470	36/50
*ChmR*	191	Flavin reductase	VlmR (AAC45645)	32/50
*Orf(+1)*	273	Chitinase	Chitinase C (1WVU_A)	58/75
*Orf(+2)*	294	Chitinase	Chitinase C (1WVU_A)	97/98
*Orf(+3)*	371	DNA polymerase III subunit beta	5AH2_A	56/58

## Data Availability

Data are contained within the article and Supplementary Materials.

## Supplementary Materials

The following supporting information can be downloaded at: https://www.mdpi.com/article/10.3390/molecules29091982/s1, Table S1: Plasmids and strains used in this study; Table S2: Primers in this study; Figure S1: Identification of three sets of homologous proteins of GrbED from *S*. sp. CB00271, using query sequences GrbE (WP_006051176.1) (A) and GrbD (WP_006051175.1) (B); Figure S2: Phylogenetic analysis of ChmQ with other known methyltransferases; Figure S3: Alignment of the adenylation domain of ChmL predicted for activation of l-graminine with other l-graminine-specific adenylation domains from megapolibactins and gladiobactin/plantaribactin; Figure S4: Phylogenetic analysis of the KS domain of ChmP with other known KSs using NaPDoS2 webtool; Figure S5: Sequence alignment of the R^0^ domain of ChmL from *S*. sp. CB00271 and *Streptomyces* sp. MNU77; Figure S6: Phylogenetic analysis of chm gene cluster from *S*. sp. CB00271 and 116 identified chm-type gene clusters from the public databases.
