# Supplementary material for: Chalkophomycin Biosynthesis Revealing Unique Enzyme Architecture for a Hybrid Nonribosomal Peptide Synthetase and Polyketide Synthase
Source: Molecules. 2024 Apr 25;29(9):1982. doi: 10.3390/molecules29091982 (PMC11085572; doi:10.3390/molecules29091982)
Supplement: Supplementary file 1 [file molecules-29-01982-s001.zip › molecules-2939550-supplementary.pdf]

Supporting Information

Chalkophomycin Biosynthesis Revealing Unique Enzyme Architecture for a Hybrid Nonribosomal Peptide Synthetase and Polyketide Synthase

Long Yang[1, 2#], Liwei Yi [3,4,#] Bang Gong[3,5], Lili Chen[2], Miao Li[3] , Xiachengcheng Zhu [3, 6, 7], Yanwen Duan[3, 6, 7], and Yong Huang [1, 2,3*]

[1]Department of Immunology, School of Basic Medical Sciences, Anhui Medical University, Hefei 230032, China.

[2]Institute of Health and Medicine, Hefei Comprehensive National Science Center, Hefei, Anhui, 230093, China;

[3]Xiangya International Academy of Translational Medicine, Central South University, Changsha, Hunan, 410013, China.

[4]University of South China,

[5]College of Pharmacy, Hunan Vocational College of Science and Technology, Changsha, 410004, China.

[6]Hunan Engineering Research Center of Combinatorial Biosynthesis and Natural Product Drug Discover, Changsha, Hunan, 410011, China.

[7]National Engineering Research Center of Combinatorial Biosynthesis for Drug Discovery, Changsha, Hunan, 410011, China.

[#]Equal contribution.

[*]Corresponding author: Prof. Yong Huang, Institute of Health and Medicine, Hefei Comprehensive National Science Center, Hefei, Anhui, 230093, China; jonghuang@ihm.ac.cn

Index

Table S1. Plasmids and strains used in this study. S3
Table S2. Primers in this study. S4
Figure S1. Identification of three sets of homologous proteins of GrbED from S. sp. CB00271, using query sequences GrbE (WP_006051176.1) (A) and GrbD (WP_006051175.1) (B). S5
Figure S2. Phylogenetic analysis of ChmQ with other known methyltransferases. S6
Figure S3. Alignment of the adenylation domain of ChmL predicted for activation of L-graminine with other L-graminine-specific adenylation domains from megapolibactins and gladiobactin/plantaribactin. S7
Figure S4. Phylogenetic analysis of the KS domain of ChmP with other known KSs using NaPDoS2 webtool. S8
Figure S5. Sequence alignment of the R^0 domain of ChmL from *S*. sp. CB00271 and *Streptomyces* sp. MNU77. .... S9
Figure S6. Phylogenetic analysis of *chm* gene cluster from *S*. sp. CB00271 and 116 identified *chm*-type gene clusters from the public databases. S10
References. S11

Table S1. Plasmids and strains used in this study.

Strains/plasmids	Descriptions	Reference/ Source
E. coli strains		
DH5α	General cloning	Commercial source
S17-1	Intergeneric conjugal transfer	Kieser T, 2000
Streptomyces strains		
CB00271	Producing strain of chalkophomycin	This study
CB00271::Δ*chmO*	*ChmO* gene replacement mutant of CB00271	This study
Plasmids		
pOJ260	*E. coli* vector, nonreplicating in *Streptomyces*, Am^R	Kieser T, 2000
pXY5001	pOJ260-based plasmid for *chmO* inactivation, Am^R	This study

Table S2. Primers in this study.

Primers	Sequences (5'-3')	Function
NRPS1.1-Up-F*	GCGGCCGCGGATCCTCTAGAGC GGCACAGGGCGAG	Construction of knockout plasmid for *chmO*
NRPS1.1-Up-R*	GAATGTGAACACACCTCGCGCA CCCC	
NRPS1.1-Tsr-F*	TGCGCGAGGTGTGTTCACATTC GAACGGTCTCTGC	
NRPS1.1-Tsr-R*	GGGCCAACTGATTTATCGGTTG GCCGCGAGA	
NRPS1.1-Dn-F*	GCCAACCGATAAATCAGTTGGC CCGCCG	
NRPS1.1-Dn-R*	ACGACGGCCAGTGCCAAGCTT CCTGCCGACGAACTCGA	
pYZNRPS1.1F	CCGCGCTTCGTCGATCTG	confirmation of mutant Δ*chmO*
pYZNRPS1.1R	TCCAGCAGCACCCCGAC	

* The red-labeled nucleic acid sequence is the overlapping region with pOJ260 and the homology arm.

Figure S1. Identification of three sets of homologous proteins of GrbED from *S.* sp. CB00271, using query sequences GrbE (WP_006051176.1) (A) and GrbD (WP_006051175.1) (B).

A

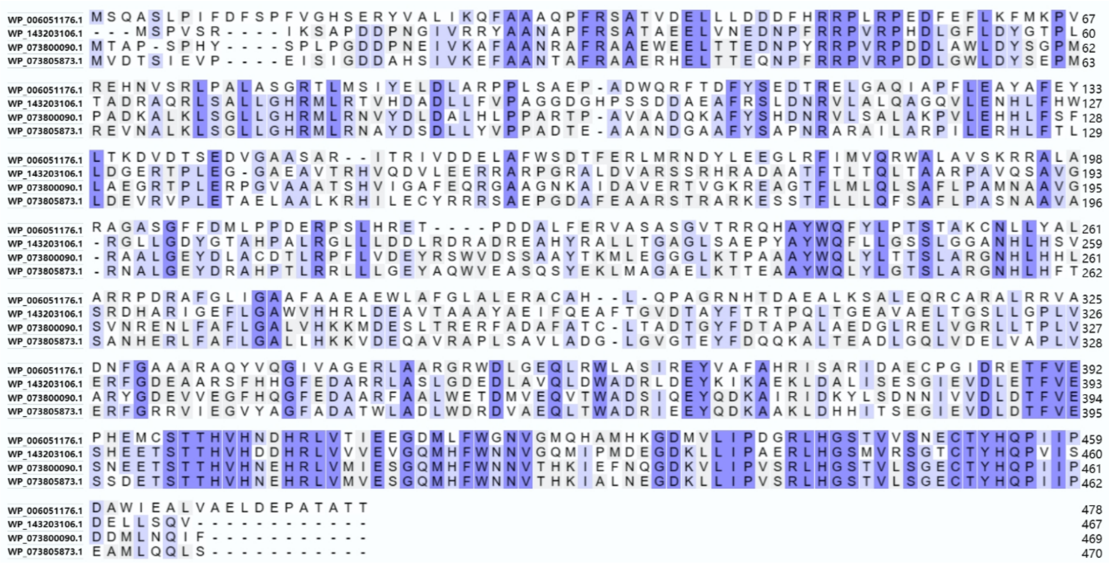

B

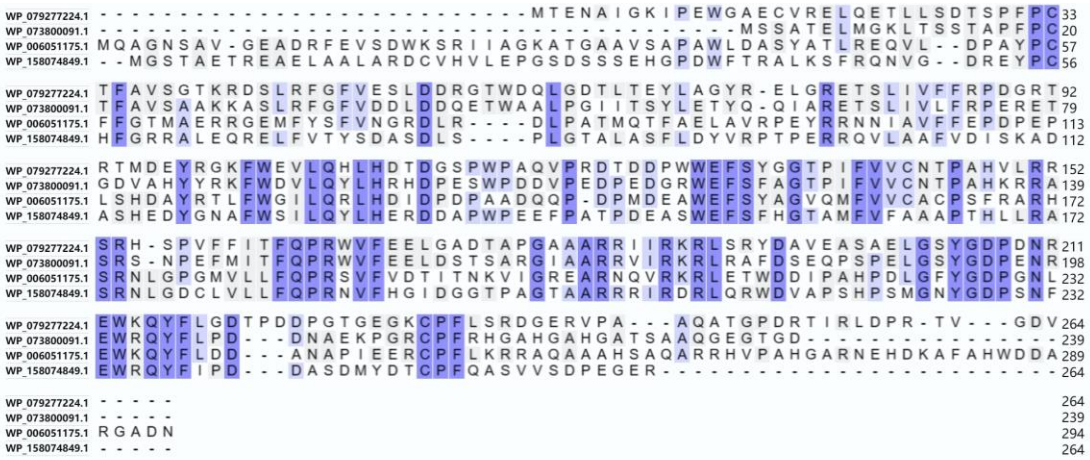

Figure S2. Phylogenetic analysis of ChmQ with other known methyltransferases.

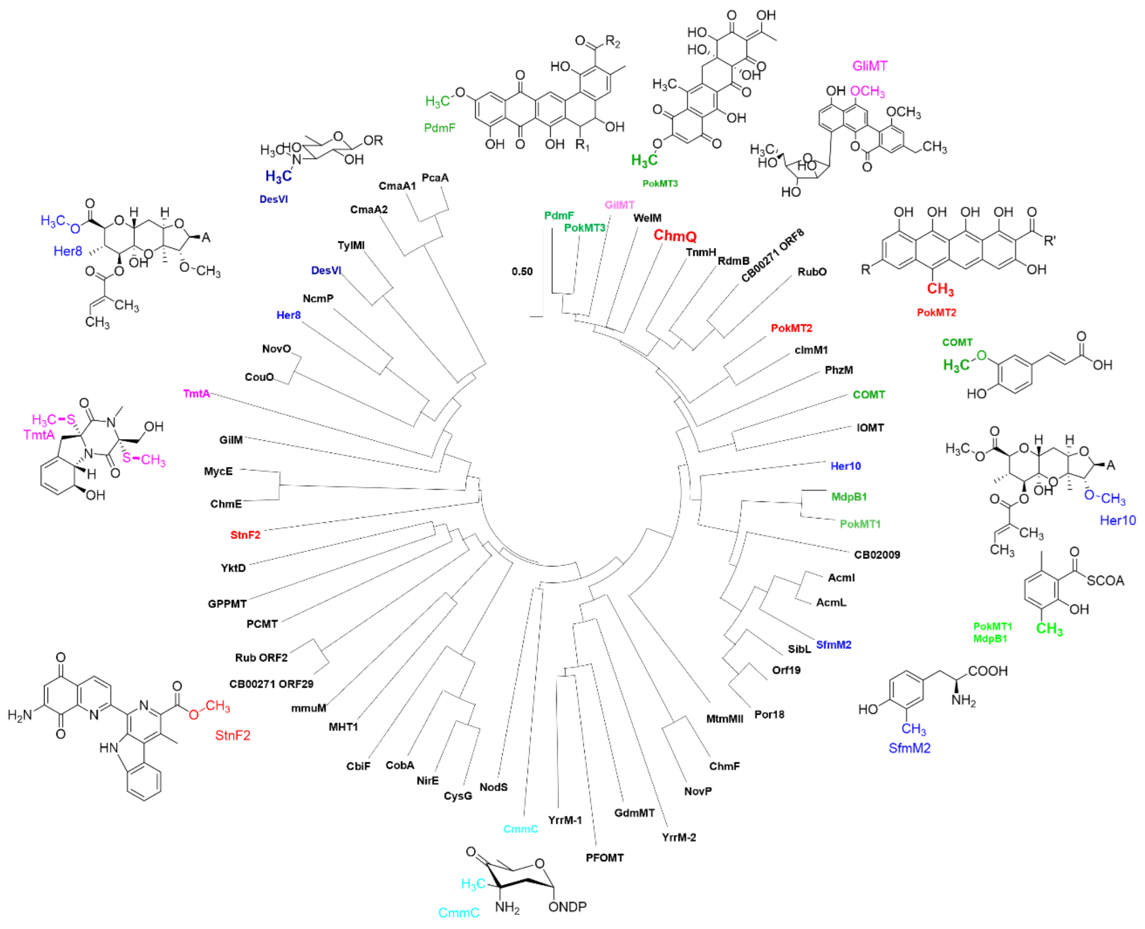

Figure S3. Alignment of the adenylation domain of ChmL predicted for activation of L-graminine with other L-graminine-specific adenylation domains from megapolibactins and gladiobactin/plantaribactin.

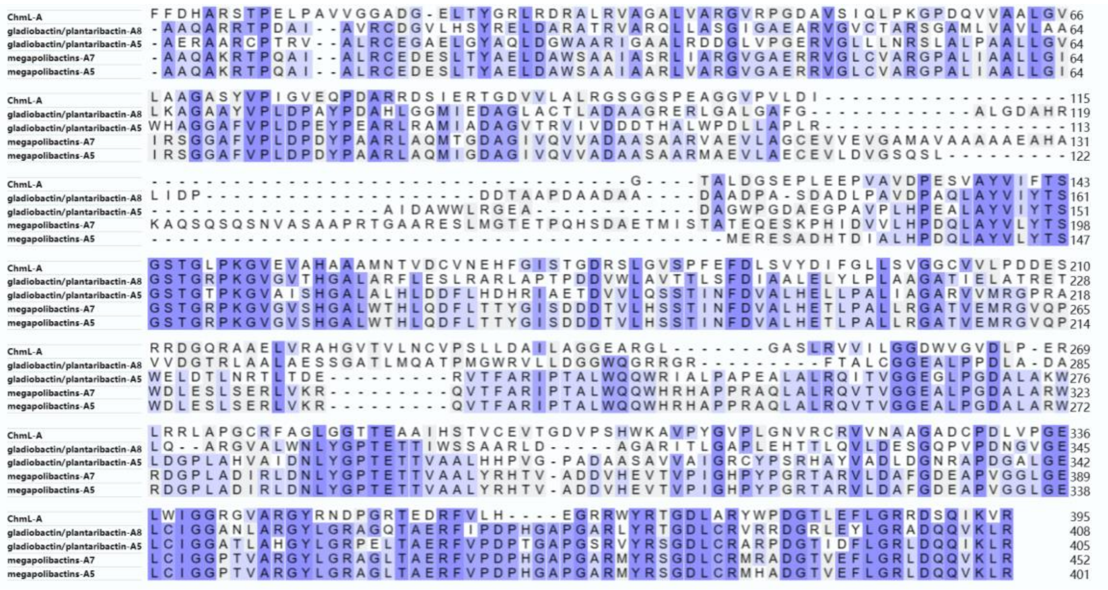

Figure S4. Phylogenetic analysis of the KS domain of ChmP with other known KSs using NaPDoS2 webtool.

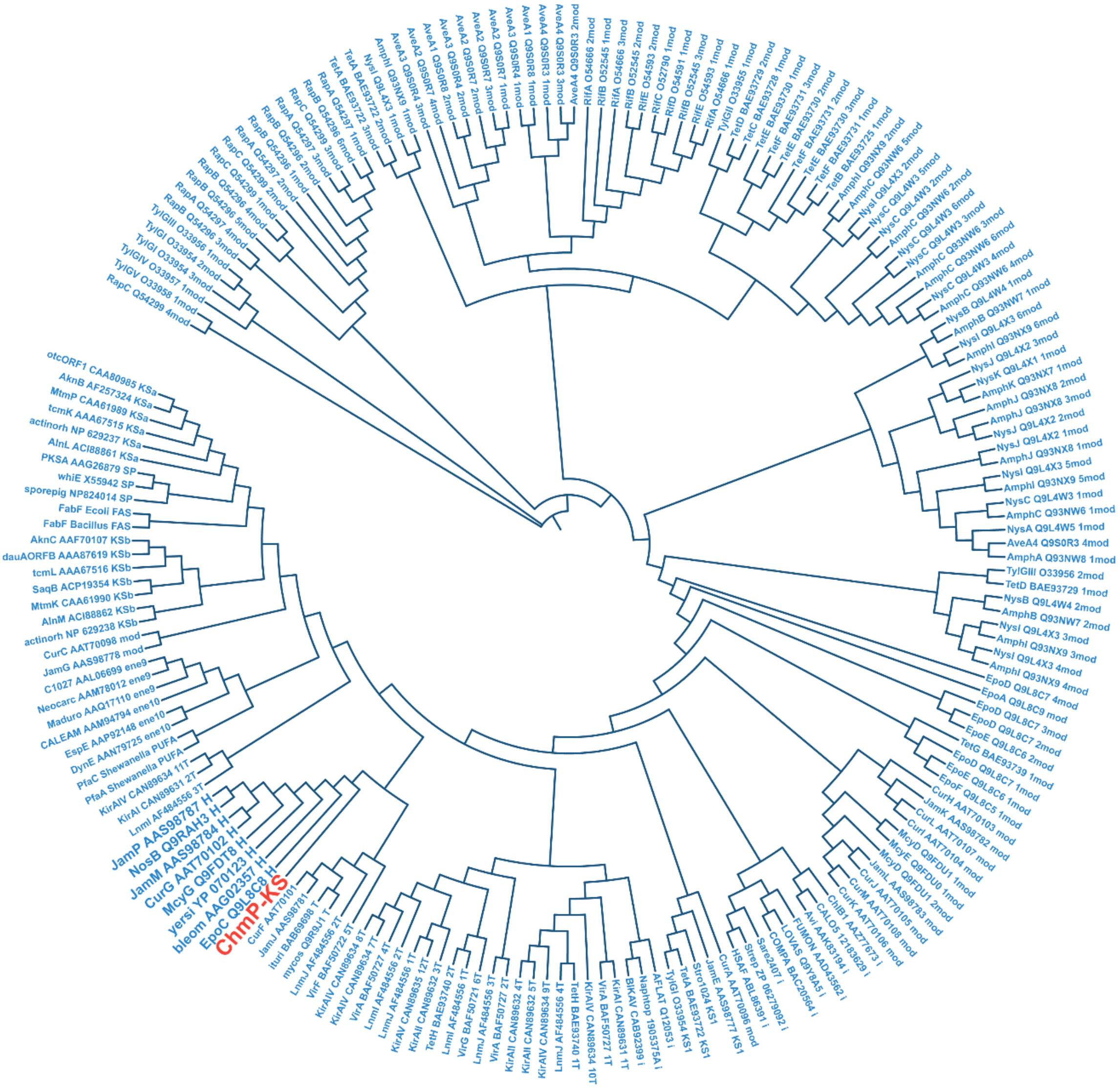

Figure S5. Sequence alignment of the R^0 domain of ChmL from *S*. sp. CB00271 and *Streptomyces* sp. MNU77.

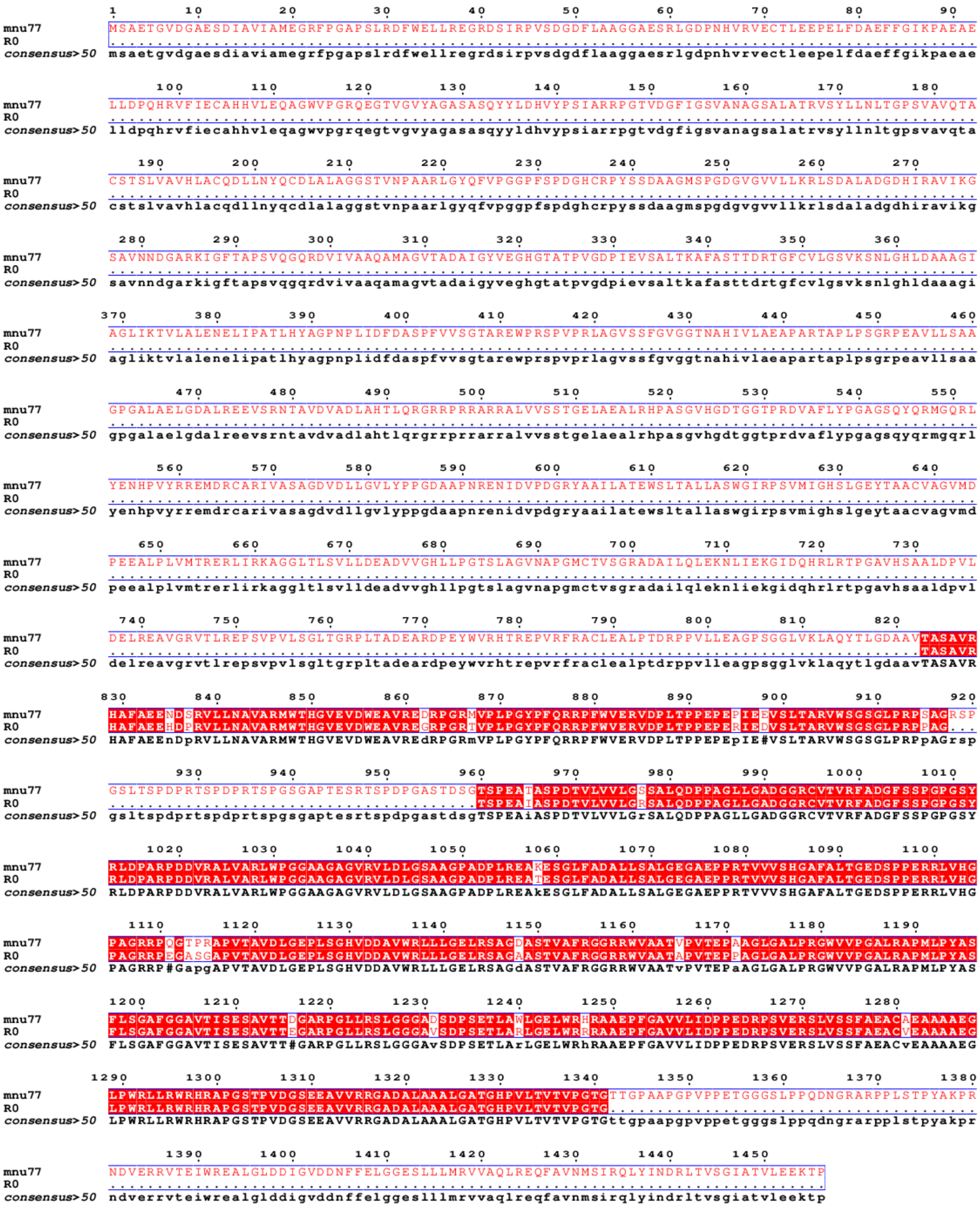

Figure S6. Phylogenetic analysis of *chm* gene cluster from *S*. sp. CB00271 and 116 identified *chm*-type gene clusters from the public databases.

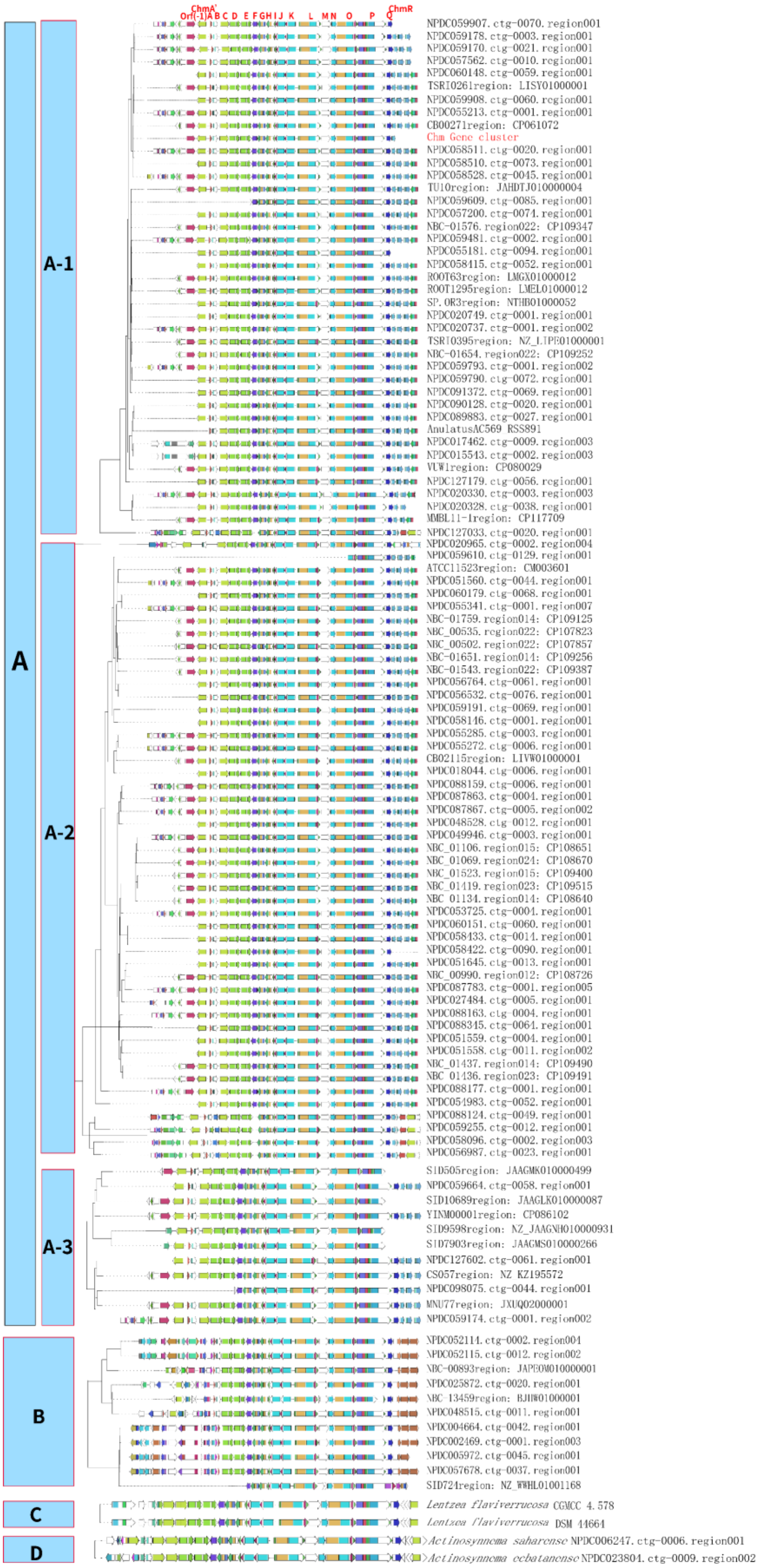

References.

1. Kieser, T.; Bibb. M.J.; Buttner, M.J.; Chater, K.F.; Hopwood D.A. (2000). Practical *Streptomyces* genetics. *John Innes Foundation, Norwich, United Kingdom.*
